# Glucocorticoid Receptor and Sequential P53 Activation by Dexamethasone Mediates Apoptosis and Cell Cycle Arrest of Osteoblastic MC3T3-E1 Cells

**DOI:** 10.1371/journal.pone.0037030

**Published:** 2012-06-14

**Authors:** Hui Li, Wenwei Qian, Xisheng Weng, Zhihong Wu, Huihua Li, Qianyu Zhuang, Bin Feng, Yanyan Bian

**Affiliations:** Department of Orthopaedic Surgery, Peking Union Medical College Hospital, Chinese Academy of Medical Science and Peking Union Medical College, Beijing, China; Rush University Medical Center, United States of America

## Abstract

Glucocorticoids play a pivotal role in the proliferation of osteoblasts, but the underlying mechanism has not been successfully elucidated. In this report, we have investigated the molecular mechanism which elucidates the inhibitory effects of dexamethasone on murine osteoblastic MC3T3-E1 cells. It was found that the inhibitory effects were largely attributed to apoptosis and G1 phase arrest. Both the cell cycle arrest and apoptosis were dependent on glucocorticoid receptor (GR), as they were abolished by GR blocker RU486 pre-treatment and GR interference. G1 phase arrest and apoptosis were accompanied with a p53-dependent up-regulation of p21 and pro-apoptotic genes NOXA and PUMA. We also proved that dexamethasone can’t induce apoptosis and cell cycle arrest when p53 was inhibited by p53 RNA interference. These data demonstrate that proliferation of MC3T3-E1 cell was significantly and directly inhibited by dexamethasone treatment via aberrant GR activation and subsequently P53 activation.

## Introduction

Glucocorticoids (GCs) are the most frequently used anti-inammatory and immunosuppressive drugs in clinic to treat a variety of diseases including inflammation, cancer, and autoimmune disorders [1]. However, it has been found that prolonged and/or overdose GCs treatment is the most common cause of osteonecrosis [2] and the third most common cause of osteoporosis [3]. It has been reported that GCs could induce apoptosis of osteoblasts and inhibit its proliferation, thus leading to osteoporosis and osteonecrosis [4]. However, the molecular mechanism of GCs involved in apoptosis and proliferation inhibition of osteoblast is still poorly understood.

The effects of GCs are primarily considered to be mediated by cytosolic glucocorticoid receptor (GR) activation [5], but the events leading from the activated GR to growth arrest are not yet elucidated completely. Previous studies have reported that GCs treatment induce osteoblast apoptosis by enhancing the expression of BH3-only protein Bim [6], down-regulation of TIMP-1 [7], and activation of glycogen synthase kinase 3 beta (GSK-3β) [8]. But to the best of our knowledge, there is no direct relationship between GR and these proteins, such as transcription-control or protein-protein interaction. We reviewed previous studies in terms of the relationship between GR activation and apoptosis, and these studies has reported that p53 [9], granzyme A [10]–[11] or Glucocorticoid-induced leucine zipper (GILZ) [12]–[13] may be the downstream molecules of GR activation.

We postulate that GC activates GR, and then leads to activation of P53, granzyme A or GILZ, thereby inducing osteoblasts apoptosis and cell cycle arrest. The results of this study indicate that GR activation indeed up-regulates the expression of P53 and its downstream molecule, which results in growth inhibition.

## Materials and Methods

### Ethical Statement

N/A.

### Reagents

Dexamethasone, and RU486 (mifepristone) were obtained from Sigma (Sigma–Aldrich Ltd, Poole, UK). Dexamethasone was used in a concentration gradient from 0.001 µM to 10 µM. Final concentration of RU486 was 10 µM. Control in all experiments was vehicle (ethanol) unless otherwise indicated. Cell counting kit (CCK-8) were obtained from Dojindo (Dojindo Molecular Technologies Inc, Gaithersburg, MD). Antibody of β-action, caspase-3, p53, PUMAand p21were purchased from cell signaling technology (CST, Danvers, MA), NOXA, granzyme A and GILZ were purchased from Abcam (Abcam, Cambridge, UK). Annexin V-FITC apoptosis determining kit were purchased from BD PharMingen (BD Biosciences, San Jose, CA). TUNEL assay was purchased from Roche Applied Science (Mannheim, Germany). Lipofectamine RNAi MAX was purchased from Invitrogen (Invitrogen Co., Carlsbad, California).

### Cell Culture

The murine osteoblastic cell line MC3T3-E1 was obtained from American Type Culture Collection (ATCC, Rockville, MD, USA). Cells were cultured in a-MEM (Gibco BRL, Gaithersburg, MD, USA) supplemented with 10% FBS, 20 mM HEPES, 100 U/ml penicillin, 100 µg/ml streptomycin, and 50 µg/ml ascorbic acid Cells were incubated in a humid incubator at 37°C (95% O_2_ and 5% CO_2_) and maintained in a subconuent state unless otherwise indicated.

### Cell Transfection

For the experiments with RNA interference (RNAi), a mouse P53 and GRα specific double-stranded, small interfering (si) RNA was synthesized (Shanghai Genepharma Co.,Ltd. Shanghai, China). Two of p53 siRNA molecules,sip53-1 and sip53-2, were selected: Sip53-1: (forward) 5- CCACUUGAUGGAGAGUAUUTT -3 and (reverse) 5- AAUACUCUCCAUCAAGUGGTT-3, and sip53-2: (forward) 5- GACCUAUCCUUACCAUCAUTT -3 and (reverse) 5- AUGAUGGUAAGGAUAGGUCTT -3. Two of GRα siRNA molecules, siGR-1 and siGR-2, were selected: siGR-1(forward) 5-GGAGAGGACAACCUGACUUTT-3 and(reverse) 5-AAGUCAGGUUGUCCUCUCCTT-3, siGR-2(forward) 5- CUGCAUGUAUGACCAAUGUTT -3 and(reverse) 5- ACAUUGGUCAUACAUGCAGTT -3.In addition, siRNA molecules that exhibited no homology to the mouse genome sequence were selected as negative controls (forward) 5- UUCUCCGAACGUGUCACGUTT -3 and (reverse) 5- ACGUGACACGUUCGGAGAATT -3 (siRNA control group, “siC”). A further untreated group of MC3T3-E1 cell cultures served as untreated control group (FBS group). Lipofectamine RNAi MAX was used to introduce the siRNA into the MC3T3-E1 cells according to the manufacturer’s instruction.

### Cell Proliferation Assay

The MC3T3-E1 cells were inoculated at 2×10^3^ cells per well in 96-well plates. To assess the effects of dexamethasone on cell proliferation, the cells were incubated in growth medium or conditioned medium for 24 h at the concentration gradient from 0.001 µM to 10 µM. The sample cells were quantified using WST-8 assay, according to the instructions. Briefly, 10 µl of 2-(2-methoxy-4-nitrophenyl)-3-(4-nitrophenyl)-5-(2,4-disulfophenyl)-2H-tetrazolium (WST-8) solution reagent was added to 100 µl of culture medium in each well. After incubation for 2.5 h at 37°C, The absorbance of each well was read at a wavelength of 450 nm on a microplate reader. The measurements were represented by the means of at least three independent experiments, with each data point based upon three replicates.

### Cell Death Detection

The MC3T3-E1Cells were seeded subconfluently into 12-well plates and treated with conditioned medium for 24 h at the concentration gradient from 0.001 µM to 10 µM. Cells were trypsinized and pelleted with cellular supernatant for 5 min at 400 g. After pellet was resuspended in 60 µL media, cell death rates were determined by counting cells using a hemocytometer after addition of Trypan blue, which stained the cytoplasm of dead cells but not live cells. Cell death rate (%) = number of dead cells/number of total cells (×100%).

### Cell Cycle Analysis

Cells were analyzed for their cell cycle distribution by flow cytometry. After being treated with dexamethasone for 48 h, the adherent cells were washed once with PBS, then trypsinized, and collected by centrifugation at 400 g for 5 min. The cells (106 cells per sample) were fixed in 4 ml of cold 70% ethanol at −20°C overnight. After centrifugation at 400×g for 5 min, cell pellets were incubated with 0.5 ml of PBS containing 100 g/µml RNase and 5 µg/ml propidium iodide (PI) at 37°C for 30 min. Cell cycle distribution was analyzed by measuring DNA content using a flow cytometer (Beckman Coulter, Inc.Fullerton, CA).

### Assessment of Apoptosis

FITC Annexin V apoptosis determining kit was used to quantitatively determine the percentage of cells within a population that are actively undergoing early apoptosis per manufacture’s instruction. Briefly, the cells were collected, washed twice with Annexin binding buffer and incubated with Annexin V-FITC and propidium iodide under the manufacturer’s recommended conditions. Cells that stain positive for FITC Annexin V and negative for PI were considered undergoing early apoptosis.

TUNEL assay was performed using the in situ cell death detection kit according to the manufacturer’s instructions. Briefly, MC3T3-E1 cells were plated onto coverslips and cultured overnight. After treated with various stress stimuli for 24 hours cells were fixed in 4% paraformaldehyde for 30 min at room temperature and permeabilized in 0.1% Triton X-100 for 2 min at 4°C. Cells were then stained with fluorescein isothiocyanate (FITC)-conjugated terminal deoxynucleotidyl TUNEL reaction mixture for 1 h at room temperature and incubated with DAB for 2 min. All dilutions were performed in PBS, and samples were analyzed and recorded by an OLYMPUS FLUOVIEW microscope.

### Real-time Polymerase Chain Reaction (PCR)

The total RNA was extracted from the cells using TRIzol reagent (Invitrogen, Carlsbad, CA, USA) and then reverse-transcribed (Promega, Madison, WI, USA) following manufacture’s protocol. The detection of P53 or GRα mRNA levels was performed by real-time RT-PCR (ABI PRISM 7900 Sequence Detection System, Applied Biosystems, Foster City, CA, USA). The primer were as follows: 5- TGTAATAGCTCCTGCATGGGG -3 (forward) and 5- TGCTATGCTTTGCTCCTGATCTG -3 (reverse) primers for P53, 5- TGCTATGCTTTGCTCCTGATCTG (forward) and 5- TGTCAGTTGATAAAACCGCTGCC -3 reverse primers for GRα, 5- ATCATGTTTGAGACCTTCAACA (forward) and 5- CATCTCTTGCTCGAAGTCCA -3 reverse primers forβ-actin. Quantification of the P53 or GRα mRNA expression was calculated using the standard curve method.

### Western Blot Analysis

Whole-cell extracts were prepared using Triton lysis buffer (50 mM Tris–HCl, pH 8.0 containing 150 mM NaCl, 1% Triton X-100, 0.02% sodium azide, 10 mM EDTA, 10 lg/ml aprotinin, and 1 lg/ml aminoethylbenzenesulfonyl fluoride). After the lysates were centrifuged for 12 min at 3,000 g to remove debris, protein concentrations were determined using the Bradford protein assay. 20–40 micrograms of protein from each cell layer homogenate was loaded onto a 10% or 12% polyacrylamide gel and transferred to a PVDF membrane. The membranes were blocked with 5% nonfat milk and incubated with primary antibodies. After extensive washing, the membranes were re-probed with peroxidase conjugated secondary antibody. Blots were processed using an ECL kit and exposed to film Controls for protein loading were made by β-actins as the internal standard.

### Statistical Analysis

All data were expressed as means ± SD. The differences between groups was analyzed by one-way analysis of variance followed by Bonferroni’s multiple comparisons test using SPSS 18.0 for windows. P-value (P) <0.05 was considered statistically significant.

## Results

### Dexamethasone Inhibit MC3T3-E1 Proliferation and Induce the Cell Death

**Figure 1:**
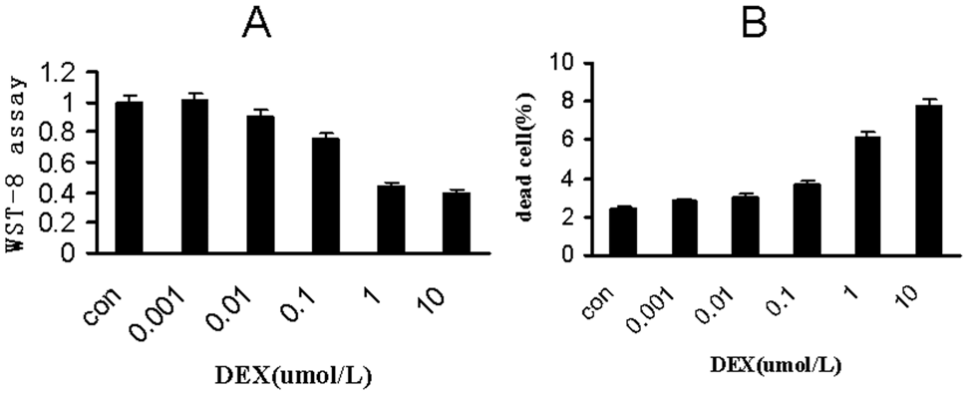
Dexamethasone dose-dependently inhibit MC3T3-E1 proliferation and induce cell death. (A) Proliferation of MC3T3-E1 cells was measured by CCK8 (colorimetric cell counting kit-8) after cells were treated with 0, 0.001,0.01,0.1, 1.0 and 10.0 µmol/L dexamethasone for 24 hours. treatment with 1 and 10 µmol/L DEX remarkably reduced cell proliferation. B) Cell death was measured by typan blue incorporation after cells were treated with 0, 0.001,0.01,0.1, 1.0 and 10.0 µmol/L dexamethasone for 24 hours. treatment with 1 and 10 µmol/L dexamethasone remarkably increased dead cell population Values are means+SEM (n = 3). *P< 0.05 vs corresponding untreated controls.

The 24-hour exposure of murine osteoblast MC3T3-E1 to 0.001, 0.01, 0.1,1.10 µmol/L DEX respectively decreased cell proliferation in a concentration-dependent manner (Figure 1A) Compared with ethanol control and low concentration dexamethasone, 1 and 10 µmol/L DEX reduced cell proliferation by 55.01±4.01% and 60.02±6.02%, respectively. To further confirm whether DEX toxicity inhibits cell proliferation, we used Typan blue incorporation to test dead cells. Typan blue assay suggested that treatment with 1 and 10 µmol/L dexamethasone remarkably increased dead cell population to 6.10±0.00% and 7.76±0.01%, respectively (Figure 1B).

### DEX-induced MC3T3-E1apoptosis and G0/G1 Arrest are Abolished by RU486 Pre-treatment

**Figure 2:**
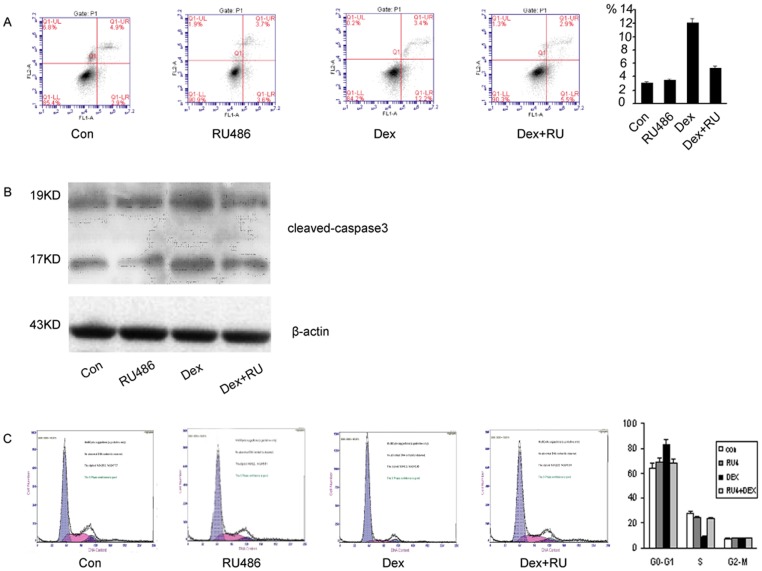
DEX-induced MC3T3-E1 apoptosis and G0/G1 arrest are abolished by RU486 pre-treatment. (A) Assessment of apoptosis in MC3T3-E1 cells using flow cytometry with Annexin V-FITC/PI staining. The apoptotic cells to total cells ratio was significantly (P<0.05) higher in DEX group compared to the control group, RU486 group, DEX+RU group. DEX+RU group and RU486 group were implemented through 2-hour pretreatment of RU486 and then DEX/ethanol treatment. (B) The level of the cleaved-caspase 3 protein was significantly up-regulated in the DEX group, when compared to that in other groups. DEX+RU group and RU486 group (C) Cell cycle analysis using flow cytometry with PI staining, showing representive histograms of MC3T3-E1 cells in control group, DEX group, RU486 group, DEX+RU group. The distribution of the cell cycle phase was expressed as the percentage of cells in the G0–G1 phase, S phase and G2-M phase of the cell cycle. The proportion of cells in the S phase decreased markedly in the DEX group in comparison to the control group, RU486 group, DEX+RU group. The proportion of cells in the G0–G1 significantly increased in the DEX group.(P<0.05) Control group: cells treated with ethnol for 24hours. DEX group: cells treated with 1 µmol/L dexamethasone for 24 hours. RU486 group: cells pre-treated 2 hours by10µmol/L RU486 and then treated with ethnol for 24 hours. DEX+RU group: cells pre-treated 2 hours by10 µmol/L RU486 and then treated with 1 µmol/L dexamethasone for 24 hours.

To determine whether the inhibitory effect can be attributed to apoptosis and cell cycle arrest, and whether the effect results from GR activation, we treated MC3T3-E1 cells with PBS (control group), 1 µmol/L dexamethasone (DEX group), RU486 (RU486 group), and 1 µmol/L dexamethasone plus 10 µmol/L RU486 (DEX+RU group) respectively. The apoptotic cells to total cells ratio was significantly (P<0.05) higher in DEX group (12.1±0.04%) as compared to 3.01±0.04% in the control group, 3.54±0.05% in the RU486 group, and 5.36±0.03% in the DEX+RU group. (Figure 2 A) The level of the cleaved-caspase 3 protein was significantly up-regulated in the DEX group, when compared to that in other groups. (P<0.05) (figure 2B). By adding RU486 2 hours prior to dexamethasone treatment, we blocked the GR and the apoptosis effect associated with GR activation.

To assess the change of cell cycle induced by dexamethasone in MC3T3-E1 cells, the cell nuclei were stained with propidium iodide and the cell cycle was analyzed through flow cytometry. The percentage of cells in the G0–G1 phase increased significantly (P<0.05) to 84.67±1.61% in the DEX group, as compared to 64.67±2.65% in the control group, 68.5.±2.65% in the RU486 group, and 68.00±0.76% in the DEX+RU group, respectively. The percentage of cells in the S phase was decreased significantly (P<0.05) to 8.90±1.00% in the DEX group as compared to 27.67±0.58% in the control group, 24.00±1.00% in the RU group, and 23.83±1.26% in the DEX+RU group, respectively. (Figure 2 C).

### GRα Gene Silencing Inhibited the G0–G1 Arrest and Apoptosis Induced by DEX

**Figure 3:**
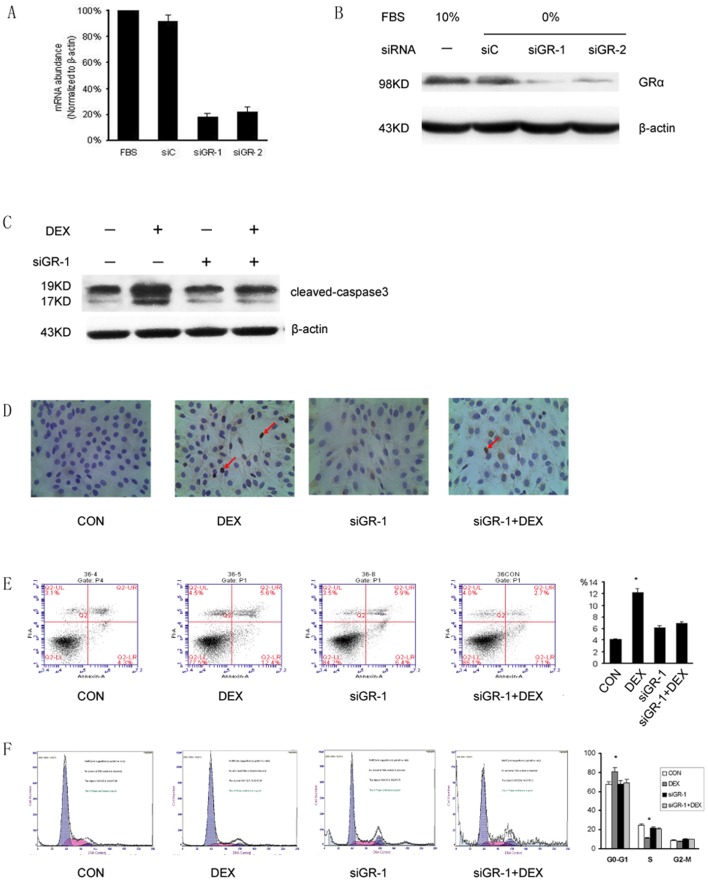
GRα Gene Silencing inhibited MC3T3-E1 G0–G1 arrest and apoptosis induced by DEX. (A) Real time PCR examination of MC3T3-E1 cells in which the GRαgene function was silenced by siRNA (siGR-1, siGR-2) targeting GRαmRNA; the mRNA expression level of GRαin the siGR-1and siGR-2 groups decreased significantly (P<0.05) compared to that in the FBS group and the siC group. (B) Examination of the protein expression level of the GRαgene by Western blotting following treatment with the indicated siRNA molecules siGR-1 and siGR-2. The protein expression level of GRαsignificantly decreased in the siGR -1, and siGR -2 groups compared to that in the siC groups and FBS groups. SiGR-1 and siGR -2 group: Cells treated with siRNA molecules siGR -1 and siGR -2, respectively. siC group: Cells treated with a siRNA which had a randomized nucleotide sequence that had no significant homology to any part of the human genome. FBS group: Cells without any treatment. (C) The level of the cleaved-caspase 3 protein was significantly up-regulated in the DEX group, when compared to that in the control group, the siGR-1 group,DEX+ siGR-1 group. (D) Visualization of apoptotic cells by the TUNEL (DAB/haemotox) assay in MC3T3-E1 cells. The cell nuclei was stained with DAPI (blue) (4′,6-diamidino-2-phenylindole).And the apoptotic cells were visualized by TUNEL staining(brown). The apoptotic cells in DEX group is significantly increased compared to the control group, the siGR-1 group,DEX+siGR-1 group. (E) Assessment of apoptosis in MC3T3-E1 cell using flow cytometry with Annexin V-FITC/PI staining. Annexin V-FITC(+)PI(−) cells were considered as early apoptotic cells. The apoptotic cells to total cells ratio was significantly (P<0.05) higher in DEX group compared to the control group, the siGR-1 group,DEX+siGR-1 group. (F) Cell cycle analysis using flow cytometry with PI staining, showing representive histograms of MC3T3-E1 cells in control group, DEX group, the siGR-1 group,DEX+ siGR-1 group. The proportion of cells in the S phase decreased markedly in the DEX group in comparison to the control group, the siGR-1 group,DEX+ siGR-1 group. The proportion of cells in the G0–G1 significantly increased in the DEX group. (P<0.05) Control group: cells treated with PBS. DEX group: cells cells treated with 1 µmol/L dexamethasone. siGR-1 group: cells treated with siRNA molecules siGR -1. DEX+ siGR-1 group: cells cells treated with 1µmol/L dexamethasone plus siRNA molecules siGR -1.

To investigate GRαgene function, we tried to silence this gene in MC3T3-E1 cells using siRNA targeting GRαmRNA. The elimination of the GRαmRNA and protein in MC3T3-E1 cell cultures was achieved as determined by real-time PCR (Fig. 3A) and Western blotting (Fig. 3B), respectively. When compared to those in untreated and siRNA control (siC) group, the mRNA and protein expression level of GRα decreased significantly (P<0.05) in the siGR-1 and siGR-2 group. On the other hand, the β-actin did not vary significantly between the groups.

To further determine the role of GR in DEX-induced apoptosis and cell cycle, we treated MC3T3-E1 cells with PBS (control group), 1 µmol/L DEX (DEX group), siGR-1 (siGR-1 group), 1 µmol/L DEX plus siGR-1 (DEX+siGR-1 group) respectively. Cleaved Capase-3 was used to characterize apoptosis of these groups. Western blot show DEX can significantly activate caspase-3 and then up-regulate cleaved Capase-3 expression, this effect can be reversed by gene silencing of GRα (Figure 3C). TUNEL staining was also performed which shows apoptotic cells increased in DEX group, this effect can abolisded by GR geen siliencing as demonstrated in the DEX+siGR-1 group (Fig. 3D). The apoptotic cells to total cells ratio was significantly (P<0.05) higher in DEX group (12.34±0.65%) as compared to 4.11±0.08% in the control group, 6.23±0.27% in the siGR-1 group, and apoptotic cell ratio decreased significantly when GRαwas silenced (6.94±0.37%). (Figure 3E).

The percentage of cells in the G0–G1 phase increased significantly (P<0.05) to 81.14±4.77% in the DEX group as compared to 67.26±3.65% in the control group, 68.4±2.65% in the siGR-1 group, and 69.34±0.76% in the DEX+siGR-1 group. The percentage of cells in the S phase was decreased significantly (P<0.05) to 11.09±0.86% in the DEX group as compared to 24.74±1.58% in the control group, 21.75±2.00% in the siGR-1 group, and 20.83±1.13% in the DEX+siGR-1 group. (Figure 3E).

### p53 but not Granzyme A or GILZ is Up-regulated by DEX Treatment

**Figure 4:**
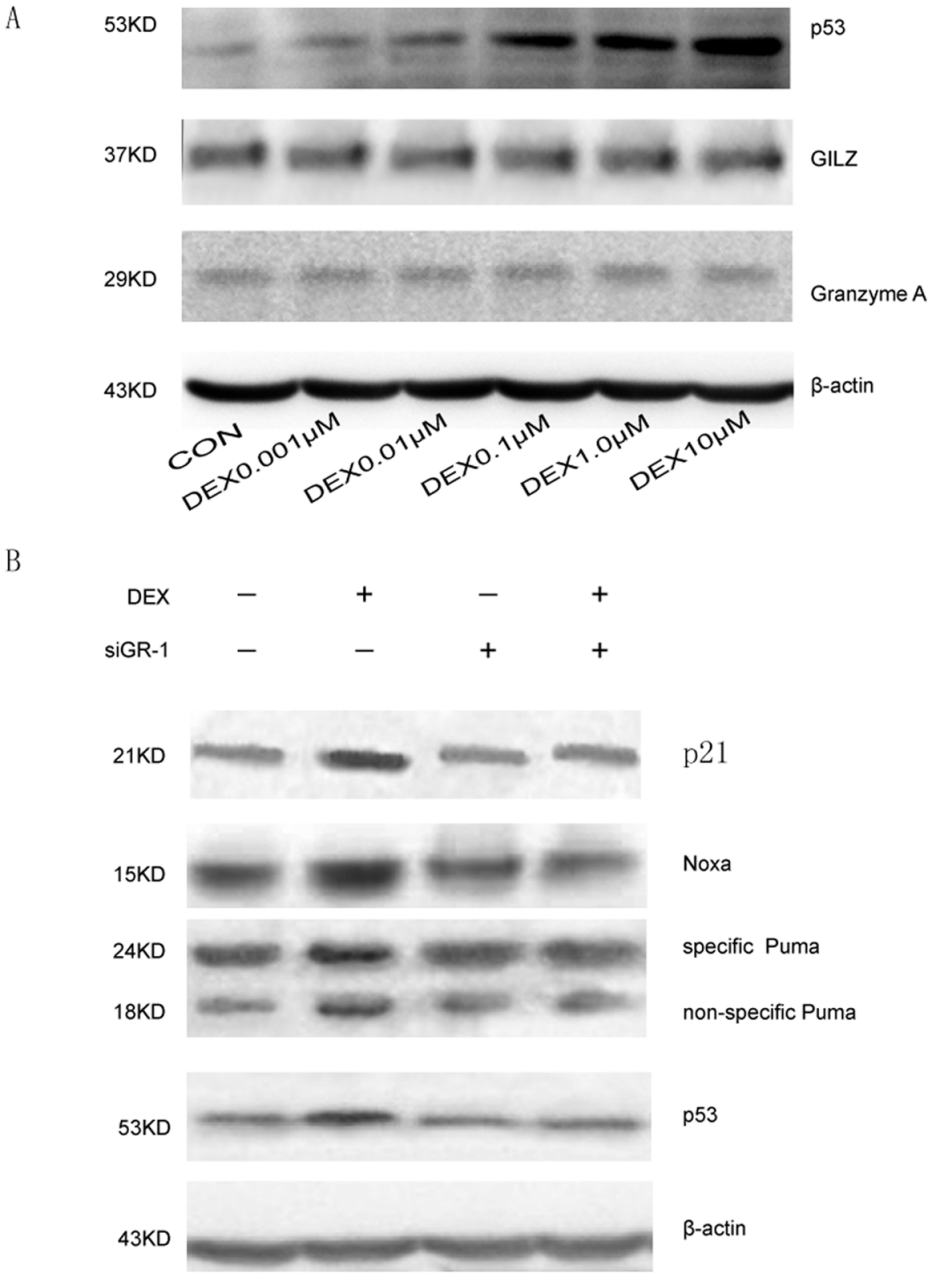
p53 but not granzyme A or GILZ is up-regulated by DEX treatment. (A) Western blot analysis to describe the levels of the proteins Granzyme A, GILZ and P53 in the MC3T3-E1 cells treated with 0, 0.001,0.01,0.1, 1.0 and 10.0 µmol/L dexamethasone for 24 hours. The levels of granzyme A and GILZ did not vary significantly between the groups, and the expression of P53 was dose-dependently up-regulated. (B) Western blot analysis to describe the levels of the proteins NOXA, PUMA, p53 and p21 in the MC3T3-E1 cells. The molecular weight markers were 53 kDa (P52), 21 kDa (p21), 18 kDa (nonspecific PUMA), 24 kDa (specific PUMA), and 15 kDa (NOXA). Control group: cells treated with PBS. DEX group: cells cells treated with 1 µmol/L dexamethasone. siGR-1 group: cells treated with siRNA molecules siGR -1. DEX+siGR-1 group: cells cells treated with 1 µmol/L dexamethasone plus siRNA molecules siGR-1.

To identify the downstream signaling pathways of GR activation, we performed Western blot analysis of proteins affected by GR activation such as granzyme A, GILZ and p53,respectively. This was done when MC3T3-E1 cells was treated with 0, 0.001, 0.01, 0.1, 1.0 and 10.0 µmol/L DEX, respectively. The levels of granzyme A and GILZ did not increased, but the expression of P53 was dose-dependently up-regulated (Figure 4A). These results suggest that P53 is a mediator of GR activation and growth inhibition.

To further confirm that and identify downstream signaling pathways of P53, we performed western blot analysis of apoptosis and cell cycle associated proteins NOXA, PUMA and p21. In the DEX group, the level of the p53 protein was up-regulated, when compared with that of the control group, siGR-1 group and siGR-1+DEX group, which was consistent with the previous results. The level of the NOXA, PUMA and P21 protein was also up-regulated in the DEX group which suggested that P53 is a key molecular between GR activation and NOXA, PUMA and P21 up-regulation. For comparison, β-actin protein was used as an internal reference, which did not vary remarkably. (Figure 4B).

### p53 Gene Silienc by siRNA can Reverse DEX Induced Apoptosis and Cell Cycle Arrest of MC3T3-E1 Cells

**Figure 5:**
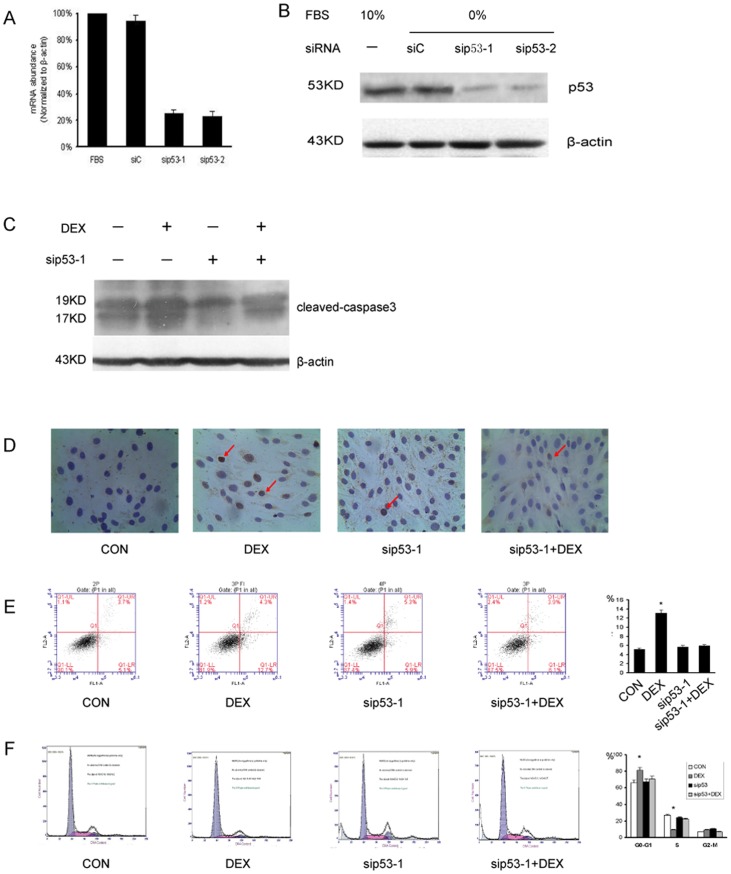
p53 gene silienc by siRNA can reverse DEX induced apoptosis and cell cycle arrest of MC3T3-E1 cells. (A) Real time PCR examination of MC3T3-E1 cells in which the p53 gene function was silenced by siRNA (sip53-1, sip53-2) targeting p53mRNA; the mRNA expression level of p53 in the sip53-1and sip53-2 groups decreased significantly (P<0.05) compared to that in the FBS group and the siC group. (B) Examination of the protein expression level of the p53αgene by Western blotting following treatment with the indicated siRNA molecules sip53-1 and sip53-2. The protein expression level of P53 significantly decreased in the sip53 -1, and sip53 -2 groups compared to that in the siC groups and FBS groups. Sip53-1 and sip53 -2 group: Cells treated with siRNA molecules sip53 -1 and sip53 -2, respectively. siC group: Cells treated with a siRNA which had a randomized nucleotide sequence that had no significant homology to any part of the human genome. FBS group: Cells without any treatment. (C) The level of the cleaved-caspase 3 protein was significantly up-regulated in the DEX group, when compared to that in the control group, the sip53 -1 group DEX+ sip53 -1 group. (D) Apoptosis as demonstarted by TUNEL (DAB/haemotox) assay in MC3T3-E1 cells. The cell nuclei was stained with DAPI (blue) (4′,6-diamidino-2-phenylindole).And the apoptotic cells were visualized by TUNEL staining(brown). The percentage of apoptotic cells in the DEX group was higher than in the control group, the sip53 -1 group DEX+ sip53 -1 group. (E) Assessment of apoptosis in MC3T3-E1 cell using flow cytometry with Annexin V-FITC/PI staining. The apoptotic cells to total cells ratio was significantly (P<0.05) higher in DEX group compared to the control group, the sip53 -1 group,DEX+ sip53 -1 group. (F) Cell cycle analysis using flow cytometry with PI staining, showing representive histograms of MC3T3-E1 cells in control group, DEX group, the sip53 -1 group,DEX+ sip53 -1 group. The proportion of cells in the S phase decreased markedly in the DEX group in comparison to the control group, the sip53 -1 group,DEX+ sip53 -1 group. The proportion of cells in the G0–G1 significantly increased in the DEX group. (P<0.05) Control group: cells treated with PBS. DEX group: cells cells treated with 1 µmol/L dexamethasone. sip53 -1 group: cells treated with siRNA molecules sip53 -1. DEX+ sip53 -1 group: cells cells treated with 1 µmol/L dexamethasone plus siRNA molecules sip53 -1.

To investigate the p53 gene function, we tried to silence this gene in MC3T3-E1 cells with siRNA targeting p53 mRNA. The knockdown of the p53 mRNA in MC3T3-E1 cell cultures was achieved as verified by RT-PCR and western blot (Fig. 5A, B). The protein expression level of p53 decreased significantly (P<0.05) in the sip53-1 and sip53-2 groups compared to that in the siRNA negative control groups and untreated groups. For comparison, the β-actin protein did not vary significantly between the groups.

We then treated MC3T3-E1 cells with PBS(control group ), 1 µmol/L dexamethasone(DEX group ), MC3T3-E1 cell silencing the p53 Gene (sip53-1 group), 1 µmol/L dexamethasone plus siRNA (DEX+sip53-1 group) respectively. Cleaved Capase-3 was used to characterize apoptosis of these groups. Western blot show DEX can significantly up-regulate cleaved Capase-3 expression, this effect can be reversed by gene silencing of p53 (Figure 5C). TUNEL staining was also performed which shows apoptotic cells increased in DEX group, but when p53 was silenced apoptotic cells decreased significantly (Fig. 5D).The apoptotic cells to total cells ratio was significantly (P<0.05) higher in DEX group (13.12±0.75%) as compared to 5.13±0.17% in the control group, 5.74±0.56% in the sip53-1 group, and this effect can abolisded by p53 geen siliencing (Figure 5E).

The percentage of cells in the G0–G1 phase increased significantly (P<0.05) to 81.44±3.56% in the DEX group as compared to 66.36±2.44% in the control group, 67.51±3.54% in the siGR-1 group,and 70.68±1.67% in the DEX+siGR-1 group. The percentage of cells in the S phase was also decreased significantly (P<0.05) to 9.54±1.07% in the DEX group as compared to 23.73±0.97% in the control group, 26.57±1.56% in the siGR-1 group, and 22.34±1.34% in the DEX+ siGR-1 group. (Figure 5E).

## Discussion

In this study, we have illustrated for the first time that GR activation by dexamethasone up-regulates P53 in MC3T3-E1 osteoblast cell line, thereby enhancing the transcriptional activatity of P53. And it ultimately induces MC3T3-E1 cells apoptosis and cell cycle arrest.

It has been reported that GCs have potent inhibitory effects on osteoblast proliferation [4]. Earlier research studies have suggested that GR activation play a pivotal role in GCs induced apoptosis [11]. In this study, we found that dexamethasone treatment not only induces MC3T3-E1 apoptosis but also arrest G1 cell cycle, which was also dependent on GR activation. We demonstrated that dexamethasone treatment can increase apoptosis more than two fold, which was consistent with previous studies [11]. However, Smith disclosed that dexamethasone arrest MC3T3-E1 at G2/M phase [14]–[15], which did not comply with our results. This may be because the culture condition in our experiments differs from that in the previous study.

The effect of GR activation by glucocorticoids has long been a research focus. It is well accepted that the effect and molecular mechanism of GR activation is cell context specific. For example, it has been reported that GR activation by dexamethasone promotes survival and inhibits apoptosis of mammary epithelial cells through transcriptional induction of serum and GC-inducible protein kinase-1 (SGK-1) and mitogen-activated protein kinase phosphatase-1 (MKP-1) [16]. On the contrary, other studies have reported that GR activation in thymocytes and lymphocytes triggers apoptosis through cathepsin B [17]. In osteoblasts, there is a consensus that glucocorticoid treatmentinduces osteoblast death both in vivo and in vitro [11], [18]. It has been reported that several important protein participate in the apoptosis or cell cycle arrest of osteoblast [6]–[8], [14] but to the best of our knowledge, there is no transcription-control or protein-protein interaction between GR and these proteins. So we need to further investigate the downstream executive molecules.

GZMA, one of the apoptotic effectors and direct transcriptional targets of glucocorticoid receptor [19], has been reported to mediate glucocorticoid-induced apoptosis of human leukemia cells [20]. Moreover, it is also reported to be involved in glucocorticoid-induced apoptosis of human osteoblast-like cells [21]. Besides GZMA, GILZ, an ubiquitous protein, whose expression is directly up-regulated by glucocorticoids, has various interaction with apoptosis and cell cycle related protein such as SGK1 and FOXO [21]–[22]. Based on the above analysis, we assumed these two protein are involved in DEX induced osteoblast apoptosis and cell cycle arrest. However, our results illustrated that DEX does not have an impact on their expression, but can significantly up-regulate the expression level of P53 and enhance its transcriptional activity as evident by elevated expression of PUMA, NOXA and p21.

The crosstalk between GR and P53 has long been a dubious topic for quite some time. Many studies verified that GR activation strengthens the transcriptional activity of p53. In a neural cell line HT-22, GR activation by DEX enhances the transcriptional activity of p53, and thus induces apoptosis and cell cycle arrest [9]. In human lung carcinoma cells, suppression of p53 blocks DEX-induced p21 (WAF1/Cip1) over-expression and G1 growth arrest [23]. In consistent with these studies, Murphy and colleagues demonstrated that the loss of p53 impairs transcription of GR target genes, and subsequently impaired GCs rescue of death in a mouse model of LPS shock [24]. These reports suggested that p53 is necessary to GR transactivation and the downstream signal transduction.

On the contrary, numerous evidence argue that there is negative cross-talk between GR and p53. Sengupta reported that GR activation resulted in p53 cytoplasmic sequestration, thus inhibiting p53’s transcriptional activity [25]. Similarly, Dex has been shown to induce ubiquitylation of GR and p53, inhibit transcriptional activity of both proteins in stressful HUVEC and normal hepatoma cells [26]. Furthermore, It has been shown that p53-mediated transcriptional activity is inhibited by GR co-expression, and that wild-type p53 efficiently inhibited GR transcriptional activity [27]. These studies confirmed that mutual inhibition between GR and p53 existed in some cell types in some specific condition.

And some other studies have also propounded that there is no relationship between p53 and GR. Dex efficiently suppressed TNF alpha-induced apoptosis through abrogated Bcl-2 reduction, which is independent of p53’s status [28]. In a similar manner, irrespective of p53 status, mammalian target of rapamycin inhibitors sensitize multiple myeloma cells to dexamethasone-induced apoptosis [29].

In this study, we have illustrated that GR activation by dexamethasone up-regulates the expression level of P53, enhances its transcriptional activity, thus resulting in p21, PUMA and NOXA up-regulation. And this subsequently induces osteoblast cell cycle arrest and apoptosis. Furthermore, we verified that disruption of p53 activation by p53 siRNA leads to the decrease in the DEX-induced cytotoxic and apoptotic activities in osteoblast cells.

The tumor suppressor p53 provides exquisite decision between cell growth arrest and apoptosis in response to various cellular stress. Sustained stress or irreparable damage trigger p53′s killer functions to initiate transcription of pro-apoptotic genes such as Puma, Noxa, Bax, and Bid. To prevent the unnecessary loss of cells which could cause premature aging, the killer functions of p53 are tightly regulated and balanced against protector functions that arrest cell cycle and support survival in response to low stress or mild damage [30]–[31]. Our results showed GR activation can up-regulate P53 expression and transcriptional activity, which paralleled with cell cycle arrest and apoptosis. While MC3T3-E1 cells (have functional GR and p53) were readily arrested in G1 phase and undergone apoptosis in response to DEX treatment, the GR-silent and p53-silent MC3T3-E1 cells preceded into S phase and survived in the presence of DEX. These results demonstrated that both GR and P53 is necessary for GC induced osteoblast apoptosis and cell cycle arrest. Furthermore, western blot results have verified transcriptional activity of p53 is dependent on GR activation.

In conclusion, our findings suggested that GR activation by DEX can up-regulate P53, which then enhances its transcriptional activatity. This mechanism contribute to apoptosis and cell cycle arrest of MC3T3-E1 cells. P53 could be used as a new target for the treatment of GC-induced osteoporosis and osteonecrosis.

## References

[1] Schacke H, Docke WD, Asadullah K (2002). Mechanisms involved in the side effects of glucocorticoids.. Pharmacol Ther.

[2] Fukushima W, Fujioka M, Kubo T, Tamakoshi A, Nagai M (2010). Nationwide epidemiologic survey of idiopathic osteonecrosis of the femoral head.. Clin Orthop Relat Res.

[3] Civitelli R, Ziambaras K (2008). Epidemiology of glucocorticoid-induced osteoporosis.. J Endocrinol Invest.

[4] Weinstein RS, Jilka RL, Parfitt AM, Manolagas SC (1998). Inhibition of osteoblastogenesis and promotion of apoptosis of osteoblasts and osteocytes by glucocorticoids. Potential mechanisms of their deleterious effects on bone.. J Clin Invest.

[5] Lowenberg M, Stahn C, Hommes DW, Buttgereit F (2008). Novel insights into mechanisms of glucocorticoid action and the development of new glucocorticoid receptor ligands.. Steroids.

[6] Espina B, Liang M, Russell RG, Hulley PA (2008). Regulation of bim in glucocorticoid-mediated osteoblast apoptosis.. J Cell Physiol.

[7] Xie H, Tang LL, Luo XH, Wu XY, Wu X (2010). Suppressive effect of dexamethasone on TIMP-1 production involves murine osteoblastic MC3T3-E1 cell apoptosis.. Amino Acids.

[8] Yun SI, Yoon HY, Jeong SY, Chung YS (2009). Glucocorticoid induces apoptosis of osteoblast cells through the activation of glycogen synthase kinase 3beta.. J Bone Miner Metab.

[9] Crochemore C, Michaelidis TM, Fischer D, Loeffler JP, Almeida OF (2002). Enhancement of p53 activity and inhibition of neural cell proliferation by glucocorticoid receptor activation.. FASEB J.

[10] Ruike Y, Katsuma S, Hirasawa A, Tsujimoto G (2007). Glucocorticoid-induced alternative promoter usage for a novel 5′ variant of granzyme A. J Hum Genet.

[11] Lu NZ, Collins JB, Grissom SF, Cidlowski JA (2007). Selective regulation of bone cell apoptosis by translational isoforms of the glucocorticoid receptor.. Mol Cell Biol.

[12] Ayroldi E, Riccardi C (2009). Glucocorticoid-induced leucine zipper (GILZ): a new important mediator of glucocorticoid action.. FASEB J.

[13] Grugan KD, Ma C, Singhal S, Krett NL, Rosen ST (2008). Dual regulation of glucocorticoid-induced leucine zipper (GILZ) by the glucocorticoid receptor and the PI3-kinase/AKT pathways in multiple myeloma, J Steroid Biochem Mol Biol 110, 244–254..

[14] Smith E, Coetzee GA, Frenkel B (2002). Glucocorticoids inhibit cell cycle progression in differentiating osteoblasts via glycogen synthase kinase-3beta: J Biol Chem.

[15] Smith E, Redman RA, Logg CR, Coetzee GA, Kasahara N (2000). Glucocorticoids inhibit developmental stage-specific osteoblast cell cycle. Dissociation of cyclin A-cyclin-dependent kinase 2 from E2F4-p130 complexes.. J Biol Chem.

[16] Wu W, Pew T, Zou M, Pang D, Conzen SD (2005). Glucocorticoid receptor-induced MAPK phosphatase-1 (MPK-1) expression inhibits paclitaxel-associated MAPK activation and contributes to breast cancer cell survival.. J Biol Chem.

[17] Wang D, Muller N, McPherson KG, Reichardt HM (2006). Glucocorticoids engage different signal transduction pathways to induce apoptosis in thymocytes and mature T cells.. J Immunol.

[18] O’Brien CA, Jia D, Plotkin LI, Bellido T, Powers CC (2004). Glucocorticoids act directly on osteoblasts and osteocytes to induce their apoptosis and reduce bone formation and strength.. Endocrinology.

[19] U M, Shen L, Oshida T, Miyauchi J, Yamada M (2004). Identification of novel direct transcriptional targets of glucocorticoid receptor.. Leukemia.

[20] Myoumoto A, Nakatani K, Koshimizu TA, Matsubara H, Adachi S (2007). Glucocorticoid-induced granzyme A expression can be used as a marker of glucocorticoid sensitivity for acute lymphoblastic leukemia therapy.. J Hum Genet.

[21] Soundararajan R, Wang J, Melters D, Pearce D (2010). Glucocorticoid-induced Leucine zipper 1 stimulates the epithelial sodium channel by regulating serum- and glucocorticoid-induced kinase 1 stability and subcellular localization.. J Biol Chem.

[22] Latre de Late P, Pepin A, Assaf-Vandecasteele H, Espinasse C, Nicolas V (2010). Glucocorticoid-induced leucine zipper (GILZ) promotes the nuclear exclusion of FOXO3 in a Crm1-dependent manner.. J Biol Chem.

[23] Urban G, Golden T, Aragon IV, Cowsert L, Cooper SR (2003). Identification of a functional link for the p53 tumor suppressor protein in dexamethasone-induced growth suppression.. J Biol Chem.

[24] Murphy SH, Suzuki K, Downes M, Welch GL, De Jesus P (2011). Tumor suppressor protein (p)53, is a regulator of NF-kappaB repression by the glucocorticoid receptor.. Proc Natl Acad Sci U S A.

[25] Sengupta S, Vonesch JL, Waltzinger C, Zheng H, Wasylyk B (2000). Negative cross-talk between p53 and the glucocorticoid receptor and its role in neuroblastoma cells.. EMBO J.

[26] Sengupta S, Wasylyk B (2001). Ligand-dependent interaction of the glucocorticoid receptor with p53 enhances their degradation by Hdm2.. Genes Dev.

[27] Zhang L, Nie L, Maki CG (2006). P53 and p73 differ in their ability to inhibit glucocorticoid receptor (GR) transcriptional activity.. Mol Cancer.

[28] Sasson R, Winder N, Kees S, Amsterdam A (2002). Induction of apoptosis in granulosa cells by TNF alpha and its attenuation by glucocorticoids involve modulation of Bcl-2.. Biochem Biophys Res Commun.

[29] Yan H, Frost P, Shi Y, Hoang B, Sharma S (2006). Mechanism by which mammalian target of rapamycin inhibitors sensitize multiple myeloma cells to dexamethasone-induced apoptosis.. Cancer Res.

[30] Schlereth K, Charles JP, Bretz AC, Stiewe T (2010). Life or death: p53-induced apoptosis requires DNA binding cooperativity.. Cell Cycle.

[31] Schlereth K, Beinoraviciute-Kellner R, Zeitlinger MK, Bretz AC, Sauer M (2010). DNA binding cooperativity of p53 modulates the decision between cell-cycle arrest and apoptosis.. Mol Cell.

